# “It’s like having a friend in your pocket.” the service user experience of the Actissist digital health intervention for early psychosis: a qualitative study

**DOI:** 10.1186/s12888-025-07071-0

**Published:** 2025-07-07

**Authors:** Sandra Bucci, Xiaolong Zhang, Daniela Di Basilio, Cara Richardson, Natalie Berry, Katherine Berry, Dawn Edge, Gillian Haddock

**Affiliations:** 1 Division of Psychology and Mental Health, School of Health Sciences, Faculty of Biology, Medicine and Health, Manchester Academic Health Science Centre, The University of Manchester, 1st Floor, Jean McFarlane Building, Brunswick Street, Manchester, M13 9PL UK; 2 Greater Manchester Mental Health NHS Foundation Trust, Manchester, UK; 3 NIHR Manchester Biomedical Research Centre, Manchester, UK; 4 Division of Health Research, Faculty of Health and Medicine, Lancaster University, Lancaster, UK

**Keywords:** Digital health intervention, Smartphone app, Psychosis, Qualitative study, Framework analysis

## Abstract

**Background:**

Understanding service users’ experience of using digital health interventions (DHIs) is essential for facilitating engagement. The Actissist app is a DHI for psychosis based on Cognitive Behavioural Therapy (CBT) principles, co-produced in collaboration with individuals with lived experience of psychosis. This qualitative study aimed to explore participants’ experiences with the Actissist app by analysing exit interviews from those who received the intervention during the Actissist 2.0 trial.

**Methods:**

Qualitative interviews were conducted with 21 participants allocated to the Actissist group in the Actissist 2.0 randomised controlled trial. Qualitative framework analysis was conducted on the interview data using a predefined sampling framework.

**Results:**

Four themes were identified. Two themes were established a priori: (1) user interaction with the app; and (2) perceived mechanism of change. Two themes were data-driven: (3) benefits of using the app; and (4) perceived barriers to app use.

**Conclusions:**

Participants viewed the Actissist app as acceptable and beneficial. Participants perceived two elements, a sense of support and normalisation and increased awareness of mental health, as the key that enabled Actissist to positively impact their mental health. Future developments of DHIs should increase the level of human support and explore the potential of adaptive sampling methods and generative AI technology to improve personalisation in frequency of prompts and content of the intervention.

**Clinical trial number:**

ISRCTN76986679, Registration Date: 07/02/2018.

**Supplementary Information:**

The online version contains supplementary material available at 10.1186/s12888-025-07071-0.

## Introduction

The use and application of mental health apps to support individuals with psychosis has grown rapidly over the past few years [1, 2]. In the last decade, there has been a remarkable expansion in the types of apps available to support individuals with psychosis, ranging from apps dedicated to symptom monitoring or psychoeducation to apps offering more comprehensive therapeutic interventions [3]. Recent studies showed that apps have the potential to enhance current care pathways providing an easily accessible, engaging addition to traditional interventions [4, 5], and can serve as stand-alone tools to inform prevention and early intervention for psychosis [6–8]. However, service user engagement over the longer term remains challenging for digital health interventions (DHIs) [9–12]. The service users’ experience of using DHIs is a key factor to improving engagement [9, 13]. Hence, it is crucial to collect and integrate user feedback in all stages of development and implementation of DHIs to meet service user needs [14–16].

The Actissist app is a DHI for psychosis based on Cognitive Behavioural Therapy (CBT) principles, co-produced by experienced clinicians, academics, computer scientists and software engineers, in collaboration with individuals with lived experiences of psychosis [17]. The core CBT section of the app, titled ‘*What’s Bothering Me*’, contains content that targets five domains associated with early psychosis relapse: perceived criticism; socialisation; cannabis use; paranoia; and distressing voice-hearing [18]. Users can also access multimedia content (e.g. videos, relaxation exercises, factsheets and web content), diary functions and customise the app aesthetics using their own images (see Supplementary Fig. 1). The treatment domains in the app can be accessed either by responding to the notifications generated by the app via pseudo-random alerts or by self-initiating use. If a user accepts a notification or initiates use, they are invited to select an intervention domain and then complete a series of self-assessment questions. The self-assessment questions are structured as question-answer exchanges that focus on cognitive appraisals, belief conviction, emotions and associated behaviours.

The DHI was tested in a proof-of-concept trial [19] and a subsequent powered RCT study (Actissist 2.0) [20]. In both trials, ClinTouch [21], a symptom monitoring app with a similar look and feel and alert schedule to the Actissist app, was utilised as the active control condition to test whether Actissist confers added benefit over and above symptom monitoring and to control for the non-specifics of using a smartphone. ClinTouch notifications prompt users to respond to a core set of items to rate the severity of 12 individual symptoms on a 1–7 scale using a touchscreen slider, with some branching items assessing further detail dependent on rating of the stem items. Symptom items have been validated against corresponding items on the Positive And Negative Syndrome Scale (PANSS) [22]. Unlike the Actissist app, self-initiated access is not available on ClinTouch, meaning data entries must be in response to a notification. The proof-of-concept trial [26] demonstrated feasibility, acceptability, safety, and potential efficacy of the Actissist app. In the exit interviews, participants expressed positive views on the Actissist app, including its ease of access, that it inspires confidence and empowerment, facilitates self-management, and that it becomes part of one’s daily routine. The powered Actissist 2.0 RCT [25] showed that using both the Actissist and ClinTouch apps improved psychotic symptoms over time with no differential effect between groups, meaning using the Actissist app did not confer added benefit on psychotic symptoms at the primary endpoint over and above symptom monitoring. This paper presents a qualitative study nested within the Actissist 2.0 trial, which explores participants’ experiences with the Actissist app and potential improvements that can be made.

## Methods

### Study setting and participants

The qualitative study was nested in the Actissist 2.0 trial [20]. The trial was approved by the West of Scotland Research Ethics Committee (REC Number: 17/WS/0221). All participants gave informed consent to participate. Participants in the Actissist 2.0 trial were registered with early intervention services (EIS) and community mental health teams throughout the Northwest of England and were deemed eligible if they: met ICD-10 criteria for schizophrenia-spectrum diagnoses; were in contact with mental health services; were within five years from onset of first psychotic episode; met a pre-determined threshold for severity of symptoms using the Positive And Negative Syndrome Scale (PANSS) [22], specifically scoring over 3 (symptom present) on any PANSS positive item and any negative or general items. Individuals who were aged less than 16 years, were not proficient in English, were participating in other psychological intervention trials, and/or were not capable of providing informed consent were excluded. The option of attending interviews was flagged at the final trial follow-up assessment. Participants were eligible to take part in the qualitative study if they had given informed consent to be interviewed about their experiences with the app and study procedures, agreed to be audio-recorded, and were not assessed as lacking capacity or experiencing current risk. A purposive sampling approach was used to ensure a diverse range of experiences and perspectives among trial participants, guided by a predefined sampling framework. The sampling framework was designed to capture variation across key demographic and engagement-related factors, including app engagement levels (e.g., high vs. low usage), age, gender, and ethnicity. This approach aimed to provide a richer understanding of the different ways in which participants interacted with and experienced the Actissist app. Of the 87 participants randomised to the Actissist group, 21 (24%) took part in the exit interview with representation across the sampling framework. While participation was voluntary, efforts were made to recruit individuals with varying levels of engagement to ensure that both frequent and infrequent users were represented. Participants were given £20 for their time and involvement in the study and reasonable travel expenses were covered.

### Procedure

Data were collected using individual semi-structured interviews informed by a topic guide (see Supplementary Table 1). The development of the topic guide was informed by the research questions, existing literature, and input from both a lived experience group and an expert reference group that comprised healthcare professionals, academics and individuals with lived experience of psychosis [17]. The framework’s a priori themes were influenced by principles from implementation science, particularly in understanding how users engaged with the app and their perceptions of its mechanisms of change. Existing research on digital mental health interventions suggests that user engagement is shaped by factors such as usability, perceived relevance, and the extent to which the intervention aligns with users’ needs and expectations [23]. Furthermore, perceived mechanisms of change and implementation frameworks highlight the importance of examining how individuals interact with digital tools and what they identify as facilitators or barriers to their effectiveness. These considerations informed the initial thematic framework, allowing for a structured yet flexible approach to data analysis. The interviews were conducted by author NB, who was a postdoctoral researcher with experience in qualitative methods. Open-ended questions were used to facilitate discussion around: (i) experiences of using the app; (ii) how it fits in with everyday life; and (iii) improvement needed. In-person interviews were conducted at a time and location preferred by the participant, usually at their home or at a clinic. Questions sequencing was dictated by the flow of the exchange. Probes were used to aid further elaboration of response.

### Theoretical framework

A framework analysis approach was utilised to analyse data, a systematic and matrix-based methodology that supports both inductive and deductive coding [24–26]. This hybrid approach was appropriate for the current study as it allowed for the incorporation of a priori themes derived from existing knowledge while also providing a structured yet flexible framework to identify and analyse additional themes that were not initially anticipated (data-driven) [26]. This approach ensured that the analysis remained grounded in established theoretical and empirical insights while allowing for the researcher to actively engage with the data for potentially new insights arising from participants’ experiences.

The analysis followed an iterative process, involving familiarisation with the data, the development of a thematic framework, indexing, charting, and mapping/interpretation. A critical realist epistemological position underpinned the research, recognising that an objective social world exists outside of people’s constructions of it, and that one’s understanding of the social world is limited by one’s experiences and position within it [27].

### Data analysis

Interviews were transcribed verbatim, anonymised and given an identifying code and stored securely. NVivo version 12 [28] was used to analyse the data. Transcripts were coded by authors CR and DDB and refined by authors XZ, SB, KB, and GH. All authors were experienced in collecting and analysing qualitative data in people with severe mental health problems. The topic guide and the outcomes of the multidisciplinary collaborative working on the Actissist project [17] were used to inform the framework’s a priori themes. We followed the proposed recursive and iterative six-stage analytical process to facilitate coding and theme-identification: (i) familiarisation with the data; (ii) generating initial codes; (iii) generating themes; (iv) reviewing potential themes; (v) defining and naming themes, and (vi) producing the study report [29]. Following the initial coding process, any data that did not fit within the a priori themes were systematically examined through an iterative and reflexive approach. Rather than assuming that themes naturally emerged from the data, we adopted an active analytical stance, recognising that theme development is a process of interpretation shaped by the researchers’ engagement with the dataset. Authors CR and DDB continuously familiarised themselves with the transcripts to become immersed with the data and used a reflective journal to reflect on the transcripts to aid interpretation of data. The additional themes were constructed through detailed review, comparison, and discussion within the research team, ensuring that patterns in the data were meaningfully organised and aligned with the study’s objectives [30]. By systematically coding and refining these themes, we ensured transparency in the analytical process while maintaining flexibility to capture participants’ experiences beyond the predefined framework. This iterative process allowed for the identification of novel insights beyond the initial coding structure, enriching our understanding of user experiences with the app. It also acknowledges the role of the researcher in actively shaping the final thematic structure. The study was reported in accordance with the Consolidated Criteria for Reporting Qualitative Research (COREQ) checklist [31] (See Supplementary Table 2).

## Results

Twenty-one participants completed an exit interview. Most participants were aged 16–25 (*n* = 11/21, 52%), male (*n* = 13/21, 62%), and white (*n* = 17/21, 81%). All participants were recruited from EIS. See Table 1 for full details of participant characteristics.

**Table 1:** Summary of participant demographics (*n* = 21)

Demographic information	*N* (%)
**Age**	
16–25 26–35 36 or older	11 (52%) 9 (43%) 1 (5%)
**Gender**	
Female Male	8 (38%) 13 (62%)
**Ethnicity**	
White Mixed Asian Black	17 (81%) 1 (5%) 2 (9%) 1 (5%)
**Service**	
Early intervention service	21 (100%)
**App Engagement**	
Low Q1 Q2 Q3 High Q4	4 (19%) 3 (14%) 5 (24%) 9 (43%)
**Phone**	
Study Own Both	13 (62%) 7 (33%) 1 (5%)

**Fig. 1:**
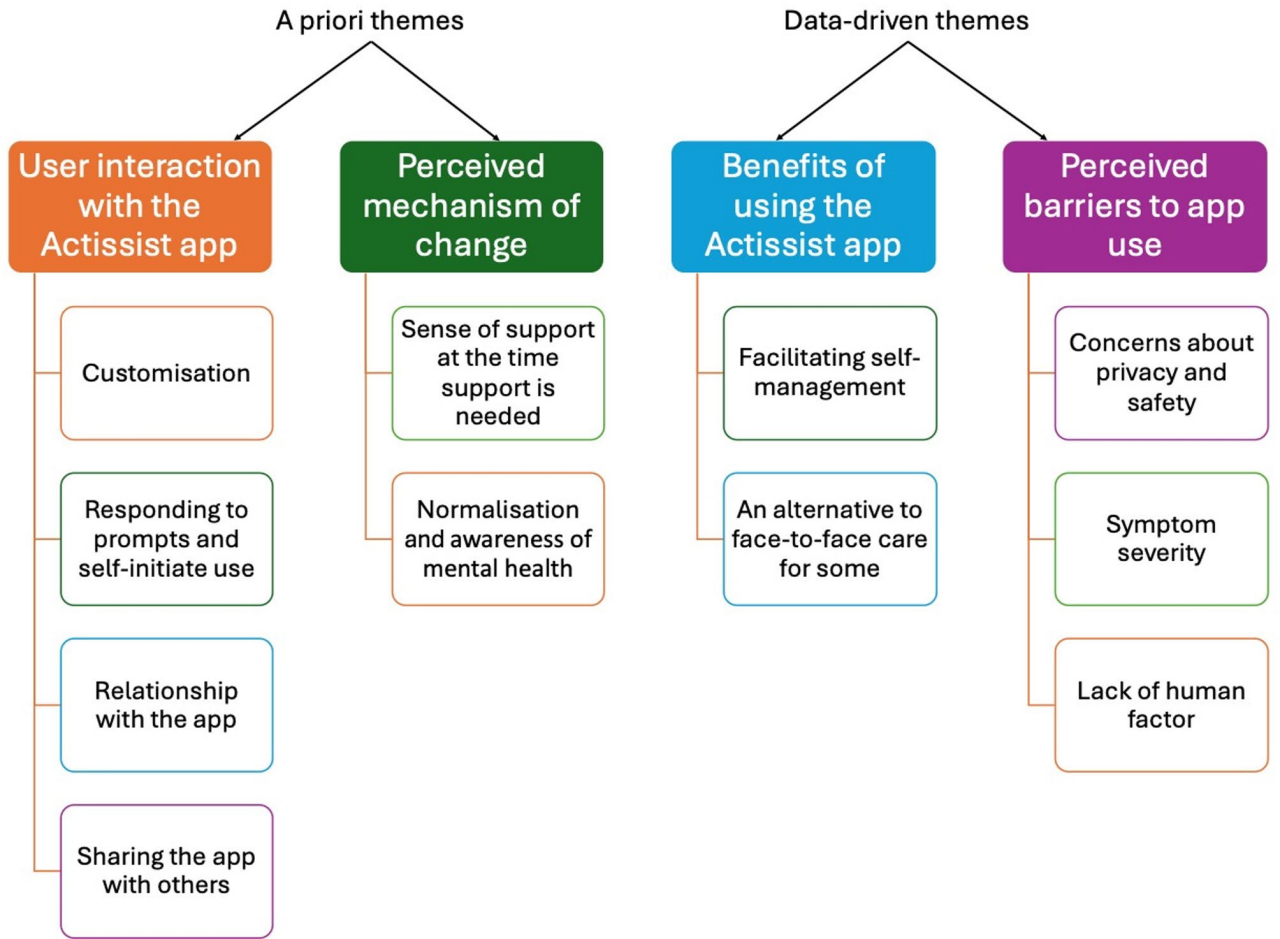
Summary of framework themes and subthemes

Two themes were established a priori: (1) user interaction with the app; and (2) perceived mechanism of change. Two themes were data-driven: (3) benefits of using the app; and (4) perceived barriers to app use. An illustrative diagram of the framework is presented in Fig. 1 and described and elaborated below.

### Theme 1: user interaction with the actissist app

Participants interacted with the app in different ways, each of which is described below.

#### Customisation

One of the strengths of the design of the Actissist app is that it allows users to customise the app interface. Some participants said they selected their own avatar and changed the wallpaper: *‘I put a picture of my dog on the wallpaper’ (Participant 115)*. This feature offered a more personal and engaging experience for participants: *‘I felt like it was just nice to go into the app and see that rather than just seeing a dull background*,* it kind of just made me smile every time I saw it’ (Participant 104)*. However, some participants chose not to use this feature, as they anticipated using the app for a short duration and therefore did not see the benefit of this feature in a DHI: *‘I think it’s because I knew I was only having it for three months and I think it’s just with apps to be honest… I think I’ve got snapchat even then I’ve not really personalised it’ (Participant 109)*.

#### Responding to prompts and self-initiated use

The main way participants interacted with the app was by responding to prompts designed to assess challenges in five core domains. Based on their answers, they engaged with corresponding CBT-informed intervention content. Most participants felt that responding to the prompts easily integrated into their daily routines, gradually cultivating a habit of responding.

*‘It’s like drinking tea*,* yeah*,* sometimes you know sitting in I take the app… it is interested to me too*,* it was part of me to you know*,* I was always waiting for the alert you know to come*,* it might come 10 o’clock in the morning*,* it was helpful’ (Participant 118).*

In terms of self-initiated use, participants reported that they would use the app when they experienced symptoms (such as hearing voices), felt distressed, had difficulty falling asleep, or wanted to relax. The domains seemed to align well with participants’ experiences, and the content delivered after selecting a target domain was both meaningful and relevant. The more relevant the content, the more participants engaged with the app.

*‘I was proper anxious because I thought people were talking about me*,* so I pressed on the thing that said ‘I feel like people are talking about me’ or ‘I’m feeling criticised’*,* and it just kind of explained to me that it would be better to just go up to the person and ask them rather than just question it*,* so I did*,* and it turned out no one was talking about me*,* so I started to use it a lot more after that’ (Participant 104).*

However, some participants noted that their usage of the app diminished as their well-being improved: *‘I guess just doing it a bit less by the end and that’s because I felt a bit better’ (Participant 102).* Due to the frequency and timing of alert notifications, participants at times ignored the app because they were otherwise engaged or had other priorities. When notifications did not interfere with other priorities, however, participants were able to engage more fully with the content.

*‘I’m sat in a room and chatting to them [friends] or chilling and watching TV and then I get a notification from the app I don’t see why the app should take priority over them over my friends’ (Participant 106).*

*‘Because it beeped during my working hours*,* so I didn’t have a lot of time to be able to delve in deep… but whilst I was at home that’s when I’d delve into more research*,* delve into more of the app and just have a play’ (Participant 121).*

#### Relationship with the app

Some participants described a ‘warming-up’ process with the app, beginning with initial doubts about its usefulness and purpose but gradually growing to appreciate its value and benefit from it.

I mean at first I thought it was kind of funny because it was just a bunch of questions that you answer but then when I started to use it a bit more it started to become clear what it actually was (Participant 104).

At first, I thought, “I’m never going to remember any of this”. I just kept thinking, “I’ll never remember it. I don’t know whether this is going to help me”, but the more times you read it, you get to know it, you get to understand it, and you can think of actions that you can take while you’re reading it, because you know what it’s about. (Participant 101)

After using it for 12 weeks, many participants felt that using the app had become part of their daily lives and over time they could see the benefits it offered.

*‘I think it grew on me over time… I think because it was there for me when I needed it*,* and I appreciated that*,* I thought you know this could be really good*,* I should try and get more out of it’ (Participant 116).*

As well as becoming integrated into their daily life, some participants noted that the regularity of using the app provided a sense of security and grounding. When they lost access to the app at the end of the treatment exposure window, they reported feeling sad and empty, and expressed that they would miss it, particularly those who lacked social connections.

*‘I miss just having the security of it*,* having it around*,* knowing that it is there*,* and I could look at it whenever I needed to’ (Participant 117).*

*‘Like I said*,* I was kind of a bit sad because I missed it*,* I’d wander around the area and there’s no app around here anymore… it was like 3 months*,* you notice when you’ve been doing something for 3 months often and then you suddenly stop… like I said*,* it checking in on me and asking how I was feeling*,* obviously I don’t usually have that because I live on my own*,* although I have been dog-sitting lately’ (Participant 116).*

In contrast, some participants viewed the app merely as a mental health management tool and felt that 12 weeks was sufficient time to take a break.

*‘Interviewer: Did you ever miss the app or miss any of the content that it gave you or anything like that?*

*Participant: Erm*,* no*,* well*,* yeah*,* it’s like at some points I always think like I wonder if I did it again would I see the results change at all or if things have improved or if things have gone worse.*

*Interviewer: Right*,* OK*,* so you*,* do you think you could use it for longer than 12 weeks then?*

*Participant: Erm*,* I think the 12 weeks was enough really*,* but then just do a break and compare the results just to see [if there will be any difference]’ (Participant 105).*

#### Sharing the app with others

Some participants shared the app with others (e.g. care team, family members, friends, or people at work) and received affirmation regarding the progress they had made. Some participants shared the app with peers facing similar challenges, which facilitated peer support.

*‘They [friends] were curious*,* and they were happy for me to be open and honest with it*,* because at the end of the day*,* I’m not afraid of being arrested or I’m not afraid of being put in front of a nurse or a doctor or a psychotherapist or any professional*,* I’ll speak my mind of what I think is right for the situation’ (Participant 121).*

*‘I show it [to] my mates as well*,* and I don’t really take drugs anymore*,* but my mates that do take drugs I shown the videos to them as well*,* and it let them get involved in what I was doing as well… they liked it… they thought it was top’ (Participant 111).*

Conversely, one participant felt it was their own responsibility to manage their mental health. They chose not to share the app with anyone other than their care team as they did not wish to burden family and friends with their mental health problems.

*“To me I don’t really talk about like me being ill anyway… especially with my family… I don’t mind talking to nurses and people who already know I’m ill… but I kind of don’t like reminding any family or friends” (Participant 109).*

### Theme 2: perceived mechanism of change

Two elements were identified as perceived facilitators of change for participants: sense of support at the time support is needed, and normalisation and awareness of mental health.

#### Sense of support at the time support is needed

Participants reported feeling a sense of support from the app as it provided useful advice on managing psychosis and was accessible at any time. This sense of connection empowered them in their self-management, boosted their confidence in recovery, and helped them feel calmer during a crisis.

*‘It’s like having a friend in your pocket… someone to give you good advice… so when your thoughts are going a bit wild you can bring it back down to earth and think about what’s happening… it talks to you’ (Participant 111).*

*‘I’m thinking every situation is going to be dangerous like taking the dog on a walk I think someone is gonna get the dog or (.) [the app tells me] it’s just your thoughts*,* you can get over that… like reading that was helping me to get over it*,* like just go out and do it gradually and badness isn’t going to happen*,* so I kept reading over that and it was really getting into my head and I was thinking ‘you can do this’’ (Participant 101).*

Most participants found that managing their mental health through an app offered convenient access to support at a time it was needed, particularly when immediate in-person appointments were not available.

*‘Yeah*,* like a relief yeah… like if I needed support*,* if it was out of hours… when I can’t phone my care coordinator*,* then I would turn to the app*,* I did have a browse*,* and I did go on it*,* you know*,* just to find a tip anywhere to like what I can do to like chill out’ (Participant 115).*

*‘Yeah*,* it was like having a boost in my pocket you know (.) like a confidence thing*,* and it was good*,* it could be accessed all the time… you could just go back and read over it again which is handy’ (Participant 101).*

#### Normalisation and awareness of mental health

Many participants felt that using the app normalised their experience of psychosis, particularly through watching videos of other’s recovery journeys. This process helped them feel less alone in managing their mental health problems, reduced the stigma associated with psychosis, and gave people hope for recovery.

*‘I thought they [the recovery journey videos] were pretty good*,* because it’s like the follow on of other people*,* and what they’ve obviously come over*,* and things like that… it was useful to see that people do overcome things*,* and they do get over it… that it can be done*,* so try stay positive’ (Participant 105).*

*‘I suppose it was positive when I was using it*,* because it would help me get out of a situation when I was like saying feeling so low*,* and I could read that and remind yourself that you know other people are doing this at the same time and you’re alright doing it and you can do it on your own (.) it can be quite personal to do’ (Participant 101).*

Participants felt that the app brought about an increased awareness of their mental health and appreciated the fact that Actissist was designed specifically for psychosis, thus offering more tailored advice compared to more general mental health and well-being focused apps. Even for those who are already familiar with psychosis, using the app served to refresh and reinforce their existing knowledge:

*‘It’s reinforced certain… knowledge from the past that may have kind of escaped me in recent months*,* so erm reacquainting myself with coping strategies and techniques I suppose’ (Participant 119).*

More importantly, the app provided insights by answering their questions and storing information for future reference (such as diary entries). This frequent reflection increased the likelihood of developing a deeper understanding of, and recognising patterns in, participant’s mental health.

*‘I think it [answering the questions] made you think deep rather than just think it was like right how am I feeling and why I am feeling like this when I am feeling like this*,* what’s going on in the situation kind of thing’ (Participant 104).*

*‘It was interesting to go back through my diary and see the days I was upset and the days when I was happy and all the entries I put in… because you forget*,* don’t you? What you were like and the frame of mind I was in when I was complaining about all my problems*,* it was just helpful you know to kind of like keep a diary’ (Participant 115).*

Increased awareness was not always perceived as a positive outcome. Some participants said that answering the questions often reminded them of their mental health challenges and the associated stigma, especially during periods when they were not particularly concerned about their mental health. This added to their emotional burden.

*‘I think after a while I was kind of like I don’t like being reminded checking in yourself and that you’re still ill… I think even though I was like ill*,* I think I was just like I’m fine*,* and it’s just like a reminder every day that it’s like ‘oh actually you have to check on your thoughts and check your behaviour’… it’s a bit tedious’ (Participant 109).*

### Theme 3: benefits of using the actissist app

Two benefits were identified: facilitating self-management, and an alternative to face-to-face care for some.

#### Facilitating self-management

Using the Actissist app encouraged users to address challenges at the time the challenge occurred rather than delaying or getting ‘caught up’ by the problem. The diverse content of the app helped participants manage a wide range of problems, fostering positive change: *‘it helps me to get out and be aware of what things were really going on’ (Participant 114)*. Furthermore, the app encouraged participants to reach out to their care team when they needed extra support, promoting help-seeking behaviours and consequently receiving timely support.

*‘There was a few tips to the voices that were saying you can control them… like speak about your problems with a professional*,* so I rang the out of hours [helpline] a couple of times… when I was there feeling particularly psychotic… I spoke to my care coordinator as well and been a lot more honest than I have been in the past… that [the app] encouraged me to be more honest*,* because they can’t help you unless they know what the problem is’ (Participant 115).*

Participants noted that the app’s goal-setting function was especially beneficial. It not only helped them set goals but also encouraged them to stay focused and continually work towards achieving those goals.

*‘[The ‘My Goals’ section of the app] it was helpful*,* because many times it reminds me of my goals*,* at the beginning*,* the lady that came that inputted it in the app*,* it reminded me of what I’m meant to do*,* what I wanted to do*,* so it was helpful… because it challenged me*,* it reminds me*,* if it doesn’t remind me*,* I won’t change’ (Participant 114).*

#### An alternative to face-to-face care for some

Some participants valued the stand-alone nature of the Actissist app, finding it less intense than face-to-face care while still offering therapeutic support. They considered it a good alternative to in-person care, as it provided a more gradual approach to sharing their feelings.

*‘If they don’t want to do therapy and that they can do the app themselves*,* it’s like*,* it’s just less full on innit*,* but it’s still got all the techniques and that and like advice’ (Participant 120).*

*‘I was proper aggressive proper closed out and isolated*,* didn’t really want to speak to no one about anything*,* and the app just kind of helped me to start slowly open up*,* even if it was just an app that I was writing to*,* it kind of made me feel a lot safer telling other people how I feel’ (Participant 104).*

### Theme 4: perceived barriers to app use

Participants reported several perceived barriers to using the app to manage their mental health: concerns about privacy and safety, symptom severity, and lack of human factor.

#### Concerns about privacy and safety

Concerns about privacy and safety when inputting information into the app significantly impacted some participants’ engagement with it. The concerns seemed less about the app’s capability to securely store information and more about the fear that the care coordinator might see sensitive details and the potential repercussions of their response. These concerns led to requests from some participants for a study phone instead of installing the app on a personal device, not using certain app functions (e.g., diary function), and reluctance to input sensitive information in the app (e.g., suicidal thoughts).

*‘If like with the whole depression side of it or if you had suicidal thoughts*,* I don’t think I would want to… I mean I don’t think I even mentioned it to my doctors or nurses if I had*,* but that was when I was very depressed… so if I knew there was a human there*,* I probably would be like no I’m not going to mention that’ (Participant 109)*.

*‘Interviewer: Did you find any of the questions hard or difficult to answer can you remember?*

*Participant: Only a couple… I only found a couple of them difficult to answer because I didn’t want to be honest to my care coordinator*,* but then I didn’t want to lie either.*

*Interviewer: Why did you not want to be honest would you say?*

*Participant: Because it was more about… like I remember it was stuff like have you harmed yourself or… do you know what I mean*,* and they ask you and you’re like ahh… what do you say back to that because you don’t want to freak them out by saying yeah*,* and you don’t want to lie by saying no*,* but then you tell them they’re going to tell your care coordinator… so it’s like loads of shit rushes through your head and you’re like… you just don’t know what to say’ (Participant 107).*

#### Symptom severity

Another frequently mentioned barrier to app engagement was the severity of participants’ mental health problems, particularly during periods when they were too unwell to use the app. During these times, using the app was seen as an additional burden, or participants simply lacked the motivation to use it.

*‘I had a lot of shit going on so… like I got locked up not long ago and I had a load of shit going on so I think it would just annoy me… I think I would smash my phone up’ (Participant 107).*

*‘I was ill at the beginning of last year and then I think I got the app a couple of months after and I don’t think I was on my antidepressants then and I was at a very low… to the point where I couldn’t even… like with the app*,* I was just pressing it and going through it and not really bother… like if someone is really low and depressed they just don’t want to do anything… like if you’ve got a three times a day reminder it’s just like I’m just going to switch my phone off’ (Participant 109).*

Participants’ engagement with the app also appeared influenced by the timing of its introduction in their recovery trajectory. For instance, if someone had recently received intensive treatment, such as in-patient care, they might not find the app as helpful in addition to the services they already received.

*‘I don’t know if that was particularly useful for me*,* and I can say if I had that at the right time during my whole psychosis journey… then I think it would be really helpful*,* and it would work towards its purpose a lot better*,* but because I had already been in hospital*,* I had met people and recovered with them… it wasn’t particularly working towards it purpose for me’ (Participant 106).*

#### Lack of human factor

Although the app’s ability to provide near real-time support was generally viewed as an advantage of the Actissist app, some participants felt that the lack of human interaction limited its benefits. They believed that the app’s limited depth of information and interaction and the need for the individual themself to make a change prevented them from fully engaging with it. This highlights the crucial role of human interaction between users and mental health professionals in providing comprehensive support and fostering meaningful change.

*‘Because it’s not a personal app*,* it doesn’t know me*,* it just knows what the input is kind of so it can’t really… obviously I don’t expect too much because it doesn’t know me*,* it only knows the input I give it*,* so it probably doesn’t know if I’m female or male’ (Participant 105).*

*‘I didn’t read all of the information that was in the app*,* because at the end of the day I’m the person that has to be empowered*,* I’m the person that has to tackle my mental illness*,* the app can’t do it for me… the app isn’t gonna stretch out arms like Inspector Gadget and give me a massive hug*,* I have to give myself a massive hug and pat on the back for doing what I’m doing’ (Participant 121).*

### Ideas for improvement

To better tailor the app to the needs of early psychosis service users, we asked participants to identify the challenges they encountered and the app’s perceived limitations. Table 2 summarises the improvements needed in response to the challenges and limitations highlighted by participants.

**Table 2:** Summary of the potential improvements needed in the actissist app

Improvement	Corresponding representative Quotes
Ongoing support on how to use the app and built-in reminders about its features	*‘Int: Do you remember going on any of the recovery journeys?* *Ppt: Erm*,* no I don’t think I seen them.* *Int: No…* *Ppt: I didn’t know there was any videos on there to be honest.* *Int: Yeah*,* so if you go onto the ‘my toolkit’ section*,* there under all them… that’s why you wouldn’t have seen them.* *Ppt: I didn’t even know about that section.* *Int: Yeah*,* so on that you’ve got your chill out area*,* your coping strategies*,* your fact sheets and the recovery journeys… so yeah that’s the additional bit… so you forgot that was there yeah.* *Ppt: Oh god*,* yeah’ (Participant 112).* *‘Yeah*,* I did use it a few times when it said like ‘how are you feeling’ you could pick whatever it was and there was one at the bottom saying other ‘do you feel the same as before’*,* which I’ve never even seen that*,* I was thinking oh they must be bored of me because I written this morning that I feel anxious and evening one I put I’m feeling anxious and I’d not seen that one where you could put the same and I’m thinking ‘they must think she’s one boring gal’ (laughing) (Participant 101)*
Reduce repetitiveness of content	*‘Ppt: Erm*,* it was OK*,* but it was a bit boring.* *Int: Right*,* OK… what makes you say that?* *Ppt: The questions*,* I was answering*,* and they just seem boring.* *Int: Any question in particular*,* or was it the repetitiveness of the questions or the actual type of questions which you found boring then?* *Ppt: It was the repetitiveness.* *Int: Right*,* OK*,* how did that make you feel?* *Ppt: Erm*,* I dunno… frustrated’ (Participant 108).* *‘After 6 or 7 weeks*,* some of the things were a bit repetitive*,* because there is only so many different things*,* because say there was two options on your toolkit*,* I tended to use about 4 or 5 of them*,* and all the other ones on the odd time*,* because I wasn’t being affected by that*,* so after a few weeks*,* it was the same things coming out again*,* and I was a bit frustrated sometimes*,* and I wanted some advice*,* and I was just getting the same advice so I was getting a bit frustrated*,* do you know what I mean’ (Participant 115).*
Expand and personalise content so that it captures more topics and is more meaningful and personalised	*‘There’s nothing on there relating to PTSD or kind of yeah dealing with that*,* and yeah*,* I don’t think there’s anything on that relating to panic attacks*,* erm*,* and I had a couple of panic attacks*,* and it’s kind of like… yeah*,* but I can see how it would help for others… I just felt like there was a bit of a gap with that side of it’ (Participant 109).* *‘I didn’t feel it helped all that much because my thing was a bit like was about death*,* and that’s where my psychosis came from that*,* and I felt like yanno on this section so what’s bothering me section getting out and about suspicious thoughts and cannabis… there was nothing kind of related to existential stuff so I kind was a bit because I was just waiting for therapy because this was just before therapy and I was kind of just like… it just seemed very limited’ (Participant 109).*
Emphasise achievements to mitigate negative effects of perceived lack of progress among users struggling to meet goals	*‘I found that [achieving the goals] hard… the ‘getting out and about’*,* because it sometimes it would remind me that I’m not getting out and about*,* and that would make me feel bad about my progress*,* and that I’ve not made any progress… but when I did get out*,* it made me feel really good’ (Participant 113).*
Fix technology difficulties	*‘Int: Right*,* okay*,* yeah… erm*,* so you remember if you went on my toolkit section then at all* *Ppt: That section didn’t end up working for me.* *Int: Oh*,* right*,* you had issues with that section.* *Ppt: Yeah*,* yeah*,* it didn’t play*,* but it did give me the idea to listen to calming music*,* like listen to my own calming music which did help’ (Participant 106).*

Int = Interviewer, Ppt = Participant

## Discussion

This study explored participants’ experiences of using the Actissist app. Four themes were identified: (i) user interaction with Actissist app; (ii) perceived mechanism of change; (iii) benefits of using the app; (iv) perceived barriers to app use. Ideas for improvement were also provided. Overall, participants found Actissist helpful, relevant, and well-suited to the mental health care pathway. Key perceived mechanisms of therapeutic change identified in the interviews included the app’s ability to provide a sense of support, helping users feel less alone in their experiences. Additionally, the app was seen as key in normalising the experience of psychosis and promoting symptom awareness, which in turn facilitated self-reflection and encouraged help-seeking behaviours. The accessibility and convenience of the app, especially for those unable to access in-person care, allows DHIs to serve as an additional tool in the help-seeking care pathway. These findings show that the user experience with Actissist aligned with its intended goal of improving access to and scaling up therapeutic support for psychosis.

Participants identified two key elements that enabled the Actissist app to positively impact their mental health: a sense of support and normalisation and increased awareness of their mental health. Given that the app was developed based on CBT principles, it is noteworthy that participants recognised normalisation, a key component of CBT, as a central element. Additionally, a sense of support and increased awareness of one’s own mental health were also reported by participants using ClinTouch in the control group of both the proof-of-concept and powered RCT studies (Trelfa et al., *under review*). The overlap in perceived mechanisms of change might explain the findings of the powered RCT study, which showed no additional benefits in the Actissist CBT-informed group compared to the ClinTouch symptom monitoring group [20]. This highlights that a sense of support and awareness of mental health appear to be key factors in facilitating change in DHIs for psychosis. This finding aligns with a review study exploring users’ experiences with DHTs in psychosis, which found that increased insight into their mental health helped participants feel more connected to their own experiences and more in control of their mental health, rather than feeling controlled by it [32].

Nevertheless, it is important to note that while increased awareness of one’s symptoms can be helpful for some, it can have the opposite effect for others, particularly when they have been feeling well. For example, when someone is feeling well, becoming aware of previous distressing experiences might lead to unnecessary worry [33]. This echoes findings from the EMPOWER study, a psychosis relapse prevention DHI, which found that heightened awareness might make some focus on potential problems or distressing memories and may increase fear of relapse, particularly when people were further along their recovery journey and the potential consequences and losses arising from a relapse increased, though the degree of fear of relapse varied across individuals [34]. Therefore, the impact of increased awareness of symptoms for some can lead to improved self-management, understanding their mental health problem and help-seeking behaviours, while for others, it might cause worry or distress, especially if they were feeling stable and well. This finding highlights the need to take precautions about using DHIs, explaining the potential harms in the consent process might be necessary [35].

Stand-alone DHIs, like the Actissist app, can be integrated into a stepped model of service delivery, offering benefits for individuals who are stable or in the early stage of their psychosis journey [12]. The non-human aspect of this tool can encourage users to gradually ‘open up’ about their mental health and experience of psychosis, especially when they are reluctant to attend in-person meetings. While the depth of stand-alone DHIs may be limited, this limitation supports the stepped model of care. DHIs can be introduced early in an individual’s journey, with more comprehensive support involving human interaction provided as needed, allowing for a step-up in care [36].

The design of the Actissist app involves prompting users to engage with assessment questions through pseudo-random alerts. However, one concern with DHIs designed this way is that the prompt can be perceived as burdensome, potentially decreasing user engagement [37]. We found that most participants felt the Actissist app seamlessly integrated into their daily lives and was especially relevant when a user was experiencing distress associated with psychosis symptoms, echoing the findings from other DHI studies in psychosis [38, 39]. While for most, the alert notifications were not overly burdensome, some participants noted that they tended to ignore the alerts when they were engaged in other priority tasks, such as at work or when they were with friends. Additionally, some participants initially expressed hesitance but gradually recognised the benefits of the app. This underscores the importance of encouraging users to persist with a DHI, even if the benefits are not immediately evident [40, 41].

Our data reinforced the concept of the digital therapeutic alliance (DTA) within DHIs as users of the Actissist app noted a sense of bond with the app. DTA refers to the relationship between a user and a DHI [42]. Inspired by the traditional therapeutic alliance in psychotherapy, it emphasises the bond between therapist and patient as crucial to achieving positive therapeutic outcomes [43]. In the context of fully automated mental health apps (i.e. apps without human support), the quality of DTA depends on the degree of flexibility, accountability, openness and emotional experiences and connections offered by an app [44]. A recent study found that goal was a core component of DTA [45], in line with traditional therapeutic alliance model conceptualised by Bordin [43]. Goal setting was highlighted as a particularly helpful feature of the Actissist app, reinforcing the CBT strategies the app drew from. However, caution is needed with this feature, as failing to achieve goals can be demoralising for participants, potentially leading to disengagement from the app. Given these findings only reflect user perceptions, more research is warranted to understand the key factors of the development and maintenance of DTA, its impact on the engagement of DHIs, and the causal link between DTA and therapeutic outcomes.

Participants reported several perceived barriers to using a standalone app like Actissist, including symptom severity, lack of human interaction, and concerns about privacy and safety. Although these barriers are commonly reported in DHI studies in psychosis [23], we found that the concerns expressed by participants around privacy did not concern the privacy of data in the app per se but were more centred around the fear of the consequence and response from mental health services. This suggests that we should provide DHI users with the option to choose whether they want to share their data and who they wish to share it with. Additionally, some participants were reluctant to share data collected by the app with family to avoid burdening them, reflecting broader concerns in mental health services about involving family in care [46]. This highlights the need for further investigation into family/carer involvement in DHIs for psychosis.

Based on the findings in this study (Table 2), we make several recommendations for improving the Actissist app that other research groups may also consider when developing their own DHI. Notably, researchers should consider developing DHIs that are agile, dynamic, and intelligent so that the system learns when to prompt users for information and when to minimize prompts. This approach, called adaptive sampling, dynamically adjusts data collection frequency [47] to maximise user engagement and experience. For example, in a mental health app, adaptive sampling might reduce the frequency of mood check-ins during stable periods and increase them during times of detected stress or instability. While modifying app content might be challenging due to the theoretical and methodological foundations of the Actissist app, the advent of large language models (LLMs) offers significant benefits. These algorithms can generate human-like text, enabling a more personalised, adaptive, and interactive user experience. LLMs can tailor responses to individual needs, provide real-time support, and adapt content dynamically based on user interactions, enhancing overall engagement and effectiveness. This advancement could help mitigate the perception of repetitive rule-based responses and promote a more dynamic form of interaction with users [48, 49].

### Strengths and limitations

Qualitative interviews afforded an in-depth examination of participants’ experiences with the Actissist app, providing valuable insights into the actual acceptability of DHIs for people with psychosis [10, 50]. There were some limitations. Due to time and financial constraints, we were not able to involve individuals with lived experience of psychosis in analysing the interviews. Participants represented a small subset of those who received the Actissist app (21 out of 87 participants allocated to the Actissist group took part in the exit interviews). Consequently, the views and experiences expressed in this study are reflective of a selection of app users and may not fully capture the diversity of perspectives of participants who used the app, and may not apply to all users of DHIs for psychosis. This introduces the possibility of selection bias, as those who chose to participate may have particularly positive or negative experiences with the app, potentially influencing the overall findings. Moreover, as participants were recruited from the Actissist 2.0 trial, our sample may have had higher digital literacy and interest in digital health compared with the broader psychosis community. Finally, the timing of exit interviews varied among participants, as they were conducted after the final follow-up time point rather than at a fixed interval following the 12-week intervention period. While efforts were made to conduct interviews as close to the intervention’s conclusion as possible, variations in timing may have influenced participants’ recall and reflections on their experiences.

## Conclusion

This qualitative study shows that the Actissist app was seen as both acceptable and beneficial by users with psychosis, aiding near real-time self-management. Participants identified that a sense of support and normalisation and increased awareness of mental health were key elements that enabled Actissist to positively impact their mental health. Improvements to the app are needed, such as ongoing support, reducing repetitiveness and offering more personalised content. These findings have important implications for enhancing the design of future DHIs for psychosis, highlighting the need for further investigation on DTA and family involvement in DHIs for psychosis. Additionally, future research should investigate the potential of blended support and utilising techniques such as adaptive sampling and LLMs to further personalise DHIs.

## Electronic supplementary material

Below is the link to the electronic supplementary material.

Supplementary Material 1

## Data Availability

The data sets generated during or analysed during this study are available from the corresponding author on reasonable request.

## Abbreviations

CBT: Cognitive behavioural therapy
CMHT: Community mental health team
COREQ: Consolidated criteria for reporting qualitative research
DHI: Digital health intervention
EIS: Early intervention for psychosis service
PANSS: Positive and Negative Syndrome Scale
RCT: Randomised controlled trial
