# Supplementary material for: “It’s like having a friend in your pocket.” the service user experience of the Actissist digital health intervention for early psychosis: a qualitative study
Source: BMC Psychiatry. 2025 Jul 7;25:682. doi: 10.1186/s12888-025-07071-0 (PMC12235868; doi:10.1186/s12888-025-07071-0)
Supplement: Supplementary file 1 — Supplementary Material 1 [file 12888_2025_7071_MOESM1_ESM.docx]

**Supplementary Figure 1.** Sample images of the Actissist app interface

**
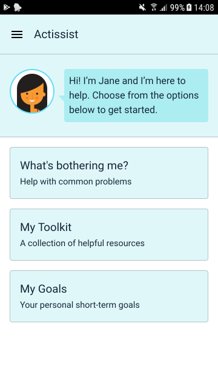
**  
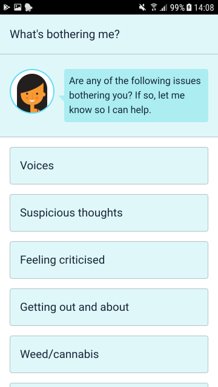
   
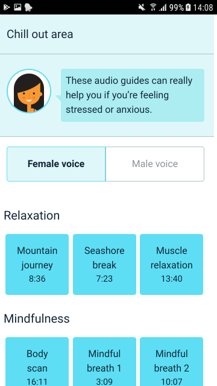


*Caption*: The home screen allows users to navigate to the core CBT section of the app (What’s bothering me?). Stand-alone multimedia content supports the feedback from the intervention domains. (e.g. patient recovery stories, mindfulness and relaxation exercises, factsheets and web content).

**Supplementary Table 1.** Topic Guide Used in Qualitative Interviews

| **What follows is a guide:** The order and exact content of the questions will be determined by the participant and will be influenced by the ongoing analysis so the order of the questions may vary as the interview develops.  *Probe and ask for examples as the time permits.* | |
| --- | --- |
| **Introduction to using the mobile phone app (Actissist):** | “Please could you describe what it was like using the Actissist app?”  ***Prompt:*** *were there any things about the app that you found particularly helpful?*  ***Prompt:*** *were there any things about the app that you didn’t find so helpful”*  “How did you feel about the app when you first saw it (i.e. at the beginning of the project)?”  ***Prompt:*** *when you first saw the app was there anything you particularly liked?*  ***Prompt:*** *when you first saw the app was there anything you particularly didn’t like or felt unsure about?*  “Have your feelings about the Actissist app changed since your first saw the app?”  ***if yes:***  ***Probe:*** *what has changed?*  “What have you learned from using the app?” ***Probe:*** what exercises or strategies in the app did you find most helpful***?***  ***Probe:*** what exercises or strategies did you find least helpful?  ***Probe:*** *did you find yourself using some of the tips suggested over and over again? Which ones?*  “Has using Actissist changed your daily life or behaviour in any way?”  ***If yes:***  ***Probe:*** *what has changed?*  ***Probe:*** *how did using Actissist help with this change?*  ***if no:***  ***Probe:*** *was there anything relating to your daily life or behaviour that you had hoped Actissist might have been able to help with?*  ***Probe:*** *why do you think Actissist didn’t help you with this?*  “Has using Actissist changed your thoughts and feelings about yourself in any way?”  ***Probe:*** *what has changed?*  ***Probe:*** *how did using Actissist help with this change?*  ***if no:***  ***Probe:*** *was there anything relating to thoughts and feelings about yourself that you had hoped Actissist might have been able to help with?*  ***Probe:*** *why do you think Actissist didn’t help you with this?*  “Has using Actissist changed your views or understanding about psychosis in any way?”  ***Probe:*** *what has changed?*  ***Probe:*** *how did using Actissist help with this change?*  ***if no:***  ***Probe:*** *was there anything relating to your views or understanding about that you had hoped Actissist might have been able to help with?*  ***Probe:*** *why do you think Actissist didn’t help you with this?*  “Do you think Actissist has helped you?”  ***If yes:*** *How?*  ***If no:*** *Why do you feel like it hasn’t helped you?*  “When we first gave you Actissist, we told you that the app is designed to help people manage some of the challenging experiences they might encounter in their day-to-day lives by using strategies informed by cognitive behaviour therapy.  “Do you think the app works for the purpose intended?”  ***If yes or no:***  ***Probe:*** *why do you think this?*  ***Probe:*** *how does/does it not meet this purpose?*  *Probe: What could we have included to help us meet our intended purpose? What suggestions do you have to make it better?*  “Do you think this is a good way to manage symptoms for people with early psychosis?”  ***If yes or no:***  ***Probe:*** *why do you think this?*  “Has using the app made any changes to the way you manage your mental health?”  ***If yes:***  *Probe: please could you tell me more about the changes you have made and why?*  “Do you think you are aware of your mood and symptoms more now than before using the app?”  ***If yes:***  ***Probe:*** *Is this a good or a bad thing? Explore…*  ***Probe:*** *could you tell me more about how you are more aware than before?*  “Has using the app changed the way in which you think about your difficulties?”  ***If yes:***  ***Probe:*** *how has it changed the way you think about this?*  “How did you feel when it came to the end of the 12-weeks using the app?”  ***Prompt:*** *did you miss Actissist when you no longer had access to it?*  ***If yes:*** *in what way did you miss it?*  ***If no:*** *is there any reason why you didn’t miss having access to the app?*  “Would you want to use the app for longer than 12 weeks?”  ***If yes:*** *why?*  ***If no:*** *why not?*  ***Probe:*** *What is the maximum length of time you think you would want to use it for?*  “Have you ever used mental health apps previously”  ***If yes:*** *what was your previous experience like? How does it compare with Actissist?*  ***If no:*** *why not?* |
| **How Actissist is used in everyday life** | “In this next part of the interview we will be talking about how you might have used Actissist in your everyday life. To help with this, I’d like you to have a look at the app now and try to remember an example of a time when you had used Actissist when you needed it.”  Are you able to think of a time when you used the what’s bothering me section on the Actissist app?  ***if yes:***  ***Probe:*** *what problem did you select on the app?*  ***Probe:*** *where were you (location) and what had you been doing at the time?*  ***Probe:*** *what made you want to use Actissist at this time?*  ***Probe:*** *can you remember what was suggested by Actissist in the hints and tips part at the end of completing the questions?*  ***Probe:*** *did you use the suggested tip?* ***If yes:*** *what happened when you used the tip?* ***if no:*** *why do you think you didn’t use the suggested tip?*  ***If client can’t think of a time when they used the what’s bothering me section:***  *That’s not a problem; it can be tricky to remember these things.*  Are you able to think of a time when you used the “My Toolkit” section on the Actissist app?  ***if yes:***  ***Probe:*** *can you remember what tab you selected in the “My Toolkit” section?*  ***Probe:*** *where were you (location) and what had you been doing at the time?*  ***Probe:*** *how did you feel about the information provided? Was it helpful?* ***If yes/no:*** *in what way?* |
| **Completing the questions and using the Multi-Media content (MMM):** | “You were given Actissist to use over a 12-week period. Did you make any changes to how you responded to the app during the time period?”  ***Probe:*** *could you tell me more about any changes in responding that you made?*  “Do you feel you had enough information to help you to use the app?”  ***If yes:***  ***Probe:*** *what kind of information have you valued the most?*  ***Probe:*** *what kind of information have you thought was least helpful?*  ***If no:***  ***Probe:*** *what kind of information would you have found helpful and why?*  “Which questions/modules were most helpful?”  ***If mention of specific questions/modules/parts of app:***  ***Probe:*** *why was this aspect of the app helpful?*  ***Probe:*** *when was this aspect of the app helpful?*  “Is there anything you would have liked to see more of (e.g. more information on psychosis, etc.)?”  ***If yes:***  ***Probe:*** *what would you like to have seen more of?*  ***Probe:*** *why would you like to see more of this? In what ways would this be helpful?*  Was there anything missing in the app that you think we should have included?  ***Elaborate…***  “Were any questions more difficult than others?”  ***If yes:***  ***Probe:*** *could you tell me which ones/what made them difficult?*  ***If no:***  ***Probe:*** *were any questions easier to answer than others?*  “Are there any questions you did not want to answer?”  ***If yes:***  ***Probe:*** *can you give any reasons why?*  “What did you think about the number of questions asked and how often they came?”  ***Probe:*** did your views on the number and how often *change over time?*  “When you engaged with the app, what were the reasons for doing so?”  “What, if anything, could we have done to make it easier for you to answer the questions?”  ***If yes:***  ***Probe:*** *could you tell me more about how and why?*  *“*How could we make each question as meaningful for you as possible?”  ***Probe:*** *did you find any questions particularly meaningful/not meaningful?*  “What could we do when you respond to questions to make responding more worthwhile or useful?”  “Did you use the app only when it beeped, or did you use it at other times during the day?”  ***Probe:*** *if you used it at other times, when did you use it/what for?*  “Did you do any of the relaxation exercises, access video clips, etc?  ***If yes:***  ***Probe:*** *Can you tell me more about that?”*  ***If no:***  ***Probe:*** *did anything stop you from using relaxation exercises/video clips etc?*  “Did you personalise your app with photographs and colour?”  ***Probe:*** *How did you personalise your app?*  “Did you refer to summary graphs?”  “Were the graphs helpful?”  ***Probe:*** *How/Why? How could we present the information in the graphs to make them more user friendly?*  “At the beginning of the project, you may remember that we sat down and set some goals you felt may be achievable during the 12 weeks that you have been using the app… what did you think about the goal setting feature on the app?”  ***Probe:*** *what did you like/dislike about the goal setting feature* |
| **Fitting in with everyday life:** | “Did you use your own phone or a study phone to access the Actissist app during the trial period? Can you think of any reasons for this?”  ***Probe:*** *was there any particular reason you didn’t use own phone/study phone?*  “How well did using the phone/app fit into your everyday life?”  ***Probe:*** *Has it changed anything that you usually do?*  “How much time did you spend using Actissist?”  ***Probe:*** *did this change at all over the 12-week period?*  “Were there any times when the beeps interrupted what you were doing?  ***If yes:***  ***Probe:*** *what was it that you were doing?*  “Have you shown it to anyone else or discussed it with anyone else? What were their views?”  ***Prompt:*** *Did you show or discuss Actissist with your care coordinator or psychiatrist?*  ***if yes:*** *was this helpful at all? What was their response like? Did this impact or change the care that you received, if yes, in what way?*  ***if no:*** *was there any particular reason why you didn’t discuss Actissist with your care coordinator or psychiatrist?*  ***Prompt:*** *Did you show or discuss Actissist with any family members or friends?*  ***if yes:*** *was this helpful at all? What was their response like? Did this impact or change the care that you received, if yes, in what way?*  ***if no:*** *was there any particular reason why you didn’t discuss Actissist with family members or friends?*  “If you were prompted to complete the questions whilst with other people, did you tell them about it?”  ***If no:***  ***Probe:*** *what did you say instead?*  “Did you feel that using the phone ever felt part of your normal routine?”  ***Probe:*** *could you tell me more about why it did/didn’t?* |
| **What could be improved:** | “How could we improve the Actissist app?”  ***Probe:*** *can you think of anything in terms of the way the app worked or the content of the app?*  “One idea for future developments is that Actissist could be linked to a wearable fitness device like a fitbit or the geolocation on your phone. This way the app could sense your location and offer tailored exercises based on your location. An example of this might be that if the phone sensed a person had not left the house in a while, the getting out an about section of the app could automatically alert the person to complete this section to help them leave the house.”  “How do you feel about the idea of Actissist linking in with your location?”  ***Probe:*** *can you think of anything that would be beneficial for you using this approach?*  ***Probe:*** *would you have any concerns about the app working in this way?*  “Are there any parts of the app you think would have been useful for you to have received an alert to use in a particular location?”  ***If yes:*** *which part of the app? Which location?*  “Privacy and Safety - did you feel that the app was safe? Did you have any privacy concerns while using the app?”  ***If yes:***  ***Probe:*** *what were you concerned about?*  ***Probe:*** *why did this concern you?*  “Did you have any difficulties using the phone or the app at the beginning of the study?”  ***Probe:*** *this could be a technical problem or something to do with content?* |
| **Any other information about Actissist not already covered** | “We’ve discussed the app in quite a lot of detail now, before we talk about your experiences of taking part in this project more generally, was there anything else you’d like to tell me specifically about your experiences of using the app? |
| **Benefits and problems:** | “Now just thinking about the project more generally…”  “Were there any benefits to taking part in the project?”  ***Probe:*** *could you tell me why they have been a benefit?*  “Were there any negative consequences for taking part in the study?”  ***Probe:*** *could you tell me why that has been a difficulty?*  “Is there anything you would change about the process?”  ***If yes***  ***Probe:*** *could you tell me more about that?*  ***Probe:*** *why would you change this?* |
| **Interview Closedown:** | “Is there anything else that you would like to tell me that we haven’t discussed, but you think might be relevant when thinking about the Actissist app or project?”  “How have you found this interview today?”  “We’ll be interviewing quite a lot of people about their experiences of being involved in the project, are there any other questions that you think might have been helpful to ask?”  “Ok I’ll now switch of the audio recorder.” |
| **End of Interview:** | Explain what will happen with the information provided.  Ask whether it would be ok to contact the participant in a few months to double check they agree with the interpretation of their information.  Ask the participant whether they would like to receive a summary of the results.  Ask the participant whether they have any questions. |

#### **Supplementary Table 2. Consolidated Criteria for Reporting Qualitative Research (COREQ) Checklist**

Consolidated criteria for reporting qualitative studies (COREQ): 32-item checklist

Developed from:

Tong A, Sainsbury P, Craig J. Consolidated criteria for reporting qualitative research (COREQ): a 32- item checklist for interviews and focus groups. *International Journal for Quality in Health Care*. 2007. Volume 19, Number 6: pp. 349 – 357.

| **No. Item** | **Guide questions/description** | **Reported on Page #** |
| --- | --- | --- |
| **Domain 1: Research team and reflexivity** | | |
| *Personal Characteristics* |  |  |
| 1. Inter viewer/facilitator | Which author/s conducted the interview or focus group? | Methods |
| 2. Credentials | What were the researcher’s credentials?  E.g. PhD, MD | Methods |
| 3. Occupation | What was their occupation at the time of the study? | Methods |
| 4. Gender | Was the researcher male or female? | N/A |
| 5. Experience and training | What experience or training did the researcher have? | Methods |
| *Relationship with participants* | | |
| 6. Relationship established | Was a relationship established prior to study commencement? | Methods |
| 7. Participant knowledge of the interviewer | What did the participants know about the researcher? e.g. personal goals, reasons for doing the research | Methods |
| 8. Interviewer characteristics | What characteristics were reported about the inter viewer/facilitator? e.g. Bias, assumptions, reasons and interests in the research topic | N/A |
| **Domain 2: study design** |  |  |
| *Theoretical framework* |  |  |
| 9. Methodological orientation and Theory | What methodological orientation was stated to underpin the study? e.g. grounded theory, discourse analysis, ethnography, phenomenology, content  analysis | Methods |
| *Participant selection* |  |  |
| 10. Sampling | How were participants selected? e.g. purposive, convenience, consecutive, snowball | Methods |
| 11. Method of approach | How were participants approached? e.g. face-to-face, telephone, mail, email | Methods |
| 12. Sample size | How many participants were in the study? | Methods |

| 13. Non-participation | How many people refused to participate or dropped out? Reasons? | N/A |
| --- | --- | --- |
| *Setting* |  |  |
| 14. Setting of data collection | Where was the data collected? e.g. home, clinic, workplace | Methods |
| 15. Presence of non- participants | Was anyone else present besides the participants and researchers? | N/A |
| 16. Description of sample | What are the important characteristics of  the sample? e.g. demographic data, date | Results |
| *Data collection* |  |  |
| 17. Interview guide | Were questions, prompts, guides provided by the authors? Was it pilot  tested? | Methods |
| 18. Repeat interviews | Were repeat interviews carried out? If yes,  how many? | N/A |
| 19. Audio/visual recording | Did the research use audio or visual recording to collect the data? | Methods |
| 20. Field notes | Were field notes made during and/or after the interview or focus group? | N/A |
| 21. Duration | What was the duration of the inter views or focus group? | N/A |
| 22. Data saturation | Was data saturation discussed? | N/A |
| 23. Transcripts returned | Were transcripts returned to participants for comment and/or correction? | N/A |
| *Data analysis* |  |  |
| 24. Number of data coders | How many data coders coded the data? | Methods |
| 25. Description of the coding tree | Did authors provide a description of the coding tree? | Results |
| 26. Derivation of themes | Were themes identified in advance or derived from the data? | Methods |
| 27. Software | What software, if applicable, was used to manage the data? | NVivo |
| 28. Participant checking | Did participants provide feedback on the findings? | Strengths and limitations |
| *Reporting* |  |  |
| 29. Quotations presented | Were participant quotations presented to illustrate the themes/findings? Was each quotation identified? e.g. participant  number | Results |
| 30. Data and findings consistent | Was there consistency between the data presented and the findings? | Results |
| 31. Clarity of major themes | Were major themes clearly presented in the findings? | Results |
| 32. Clarity of minor themes | Is there a description of diverse cases or discussion of minor themes? | Discussion |
